# Understanding dosage effects of traditional Chinese medicine using network analysis

**DOI:** 10.3389/fphar.2025.1534129

**Published:** 2025-05-08

**Authors:** Jiawei Wu, Dianjing Guo

**Affiliations:** State Key Laboratory of Agrobiotechnology and School of Life Sciences, The Chinese University of Hong Kong, Shatin, Hong Kong SAR, China

**Keywords:** dosage effect, network analysis, drug mechanism, traditional Chinese medicine, algorithm optimization

## Abstract

**Background:**

Traditional Chinese Medicine (TCM) prescriptions are complex, multi-botanical drug systems in which dosage critically influences therapeutic efficacy. While network pharmacology is widely used to analyze TCM mechanisms, existing methods ignore the dosage of botanical drugs, a key limitation that may skew predictions. This study investigates how integrating dosage data alters network analysis outputs, addressing a fundamental gap in understanding TCM’s dosage-dependent effects.

**Methods:**

Our analysis compared dosage-weighted and traditional non-dosage network approaches across 94 traditional Chinese medicine (TCM) prescriptions. We developed four custom indicators to quantify differences throughout the network pipeline: Dedis (input distance difference), DeSD (input standard deviation difference), DeDT (drug target prediction difference), and DePy (pathway prediction difference). The interrelationships among these indicators were examined to indicate when dosage adjustments influence predictions. A detailed case study further demonstrated the impact of dosage modifications on predictive outcomes.

**Results:**

Among the indicators with inputs difference, Dedis, but not DeSD, exhibited a statistically significant relationship with output predictions, with target differences (DeDT) ranging from 0% to 68.9% and pathway differences (DePy) ranging from 0% to 74.6%. The interrelationships between these indicators were visualized using a clock model representation. The case study further demonstrated the impact of dosage on network outputs, revealing dosage refined both the predicted drug targets for individual botanical drugs and the subsequent pathway analysis results.

**Conclusion:**

Our study demonstrated that dosage significantly influences the outcomes of network analysis, with Dedis serving as a reliable indicator of whether such changes would occur. Specifically, changes resulting from dosage-dependent refinement of both drug target prediction and pathway analysis were observed.

## 1 Introduction

The efficacy of drug combinations is highly dependent on the dosage of their components (Suberu et al., 2013; Xu et al., 2018; Yi et al., 2022). This principle also applies to Traditional Chinese Medicine (TCM), where prescriptions function as “super-combinations” comprising multiple botanical drugs. In TCM practice, dosages are meticulously adjusted based on patient-specific factors such as age, gender, and symptoms to achieve personalized therapeutic outcomes (Zha et al., 2015). Despite the clinical importance of dosage, mechanistic studies of TCM face significant challenges due to the complexity of its multi-botanical drug formulations, their intricate interactions, and the dynamic interplay between metabolites of botanical drugs and biological systems.

To address these challenges, computational approaches, e.g., network pharmacology, emerged as powerful tools for deciphering TCM mechanisms (Bulusu et al., 2016; Cheng et al., 2019; Nogales et al., 2022). This method aligns with TCM’s holistic philosophy by integrating data on botanical drugs, bioactive metabolites, molecular targets, and diseases into one network (Hopkins, 2008; Li, 2009). By mapping these relationships, network analysis can identify functional molecules and potential targets within a prescription, offering valuable insights for experimental validation. However, while advancements have been made in data quality, methodology, and interpretation, existing network analysis has consistently overlooked a critical factor: the dosage of botanical drugs (Li et al., 2022; Zhao et al., 2023; Xin et al., 2021; Jiashuo et al., 2022).

In this study, we systematically evaluated the impact of dosage on network analysis predictions. Using a dataset of 94 TCM prescriptions, we compared the prediction outcomes from traditional (non-dosage) and dosage-weighted network analysis. By establishing several quantitative observation indicators, dosage influence on these predictions was rigorously assessed. Finally, a case study was used to demonstrate how dosage-weighted network analysis can elucidate the mechanistic basis of a TCM prescription. Our findings highlight the indispensable role of dosage in understanding the mechanism of combination drugs.

## 2 Materials and methods

### 2.1 Traditional prescription collection and filtering

The data used in this study were derived from our previous research, which systematically analyzed TCM prescriptions to identify potential drug candidates for the treatment of pox (Wu and Guo, 2024). The prescriptions were manually collected from online databases and local libraries to ensure comprehensive coverage. All data are publicly available and accessible through our prior publication. Given that this study focused on the dosage effect in TCM analysis, two types of prescriptions were excluded: 1. Prescriptions containing botanical drugs with non-quantifiable dosage units, such as “50 grains of glutinous rice,” “two dates,” or “a handful of bamboo leaves.” 2. Botanical drugs for which relevant information—specifically bioactive metabolites and targets—could not be retrieved using the TCM-LTM database (Li et al., 2022).

### 2.2 Retrieval of network components

The network components for analyzing TCM include botanical drugs, their bioactive metabolites, the targets of these metabolites, and the pox viruses along with their associated targets. To retrieve the metabolites and their corresponding targets, the LTM-TCM database was utilized. The LTM-TCM database specifically designed for network pharmacology research in TCM, is comprised of data from 14 authoritative databases, encompassing information on 9,122 botanical drugs, 34,967 metabolites, and 13,109 targets (Li et al., 2022). The bioactive metabolites within the database are defined based on Lipinski’s rule. Using this resource, we collected the bioactive metabolites and their corresponding targets for each of the 94 prescriptions.

Although pox viruses are diverse, they exhibit a degree of homology. Four prevalent species including vaccinia, smallpox, chickenpox, and mpox were selected for this study. A total of 686 disease-associated target genes were identified from the GeneCards database (Stelzer et al., 2016) using these four pox viruses as search keywords.

### 2.3 Network building

In this study, the network was constructed based on the “botanical drugs–bioactive metabolites–target–pox virus” framework. In the unweighted network, each relationship was treated equally, and a weight of one was assigned to the edges connecting the components of the network. For the dosage-weighted network, the dosage of each botanical drug was used to assign weights to the corresponding edges. The key step in this process involved constructing a standardized dosage vector, which was derived by standardizing the dosages of botanical drugs within the prescriptions. For example, in the prescription called Chong He powder, the components included one Fen of Bai Yao Zi, one Fen of Gan Cao, and one Qian of Xiong Huang. Using the unit conversion relationships (Qiu, 1996) summarized in Table 1, these traditional units were converted into metric units, resulting in a dosage vector of [0.4 g, 0.4 g, 4 g], which was then normalized by dividing each value by the minimum weight (0.4 g in this case) to yield a standardized dosage vector of [1, 1, 10]. The standardized dosage vector was subsequently applied as the weight to the edges connected to the corresponding botanical drugs. In contrast, in the non-dosage network, the standardized dosage vector for this prescription was [1, 1, 1].

**TABLE 1:** The unit conversion in four dynasties.

	Song-Yuan (960–1,368)	Ming-Qing (1,368–1,911)
Metric conversion	1 Fen = 0.4 g	1 Fen = 0.37 g
Conversion of units	1 Liang = 10 Qian = 100 Fen	

### 2.4 The output of the network

For both the non-dosage and dosage-weighted networks, nodes with a degree higher than the average were identified as key targets of the prescription. These key targets were subsequently compared to pox-related targets, and the overlapping targets were defined as drug targets for the prescription. To explore the potential working mechanisms of the prescriptions, the version of 4.14.4 clusterProfiler R package (Wu et al., 2021) was employed for Gene Ontology (GO) annotation based on the filtered drug targets. A significance threshold of p < 0.05 and a minimum of 10 enriched genes associated with each pathway were applied to identify biologically relevant pathways, which provides insights into the potential mechanisms underlying the therapeutic effects of the prescriptions.

### 2.5 The output difference

The output of the network analysis included the predicted drug targets and their corresponding pathways. To quantify the differences in output between the non-dosage and dosage-weighted networks, two indicators were created: 1. Difference in Drug Targets (DeDT) to measure the disparity between the predicted drug targets from the two networks; 2. Difference in Pathways (DePy) to quantify the variation in the enriched pathways associated with the predicted drug targets.

$$DeDT\;or\;DePy=1-\frac{S_{1}\cap S_{2}}{S_{1}\cup S_{2}}$$

The 1-Jaccard Similarity Index (Kosub, 2019) was employed to quantify the difference. The set of drug targets or pathways predicted by the non-dosage network was denoted as S_1_, while S_2_ represents the corresponding predictions from the dosage-weighted network.

### 2.6 The input difference

The input to the network consisted of botanical drugs and their associated metabolites.

#### 2.6.1 The difference in distance (Dedis)

We used Euclidean distance to measure the distance between two points in Euclidean space (Tabak, 2014). Here, it measured the distance between the standardized dosage vector and the unweighted vector. The calculation formula was as follows.

$$Dedis=\sqrt{\sum_{i=1}^{n}{\left(x_{i}-y_{i}\right)}^{2}}$$

Where x and y are a standardized dosage vector, where x is derived from the dosage-weighted network and y is derived from the corresponding non-dosage network.

#### 2.6.2 The difference in standard deviation (DeSD)

The standard deviation (SD) was used to measure the degree of stability within the system. The dosages of botanical drugs in the prescription formed the dosage system. Since the ratio of each component was equal to one in the unweighted network, the standard deviation was always 0. Therefore, the SD of the input in the dosage-weighted network was the difference in the stability of the two networks. Here, we used the sd() function of R_4.4.1 to calculate the SD of the dosage vectors for botanical drugs and bioactive metabolites, respectively.

### 2.7 Statistical analysis

Wilcoxon rank-sum test was utilized to assess the statistical significance of relationships between network input and output variables, as well as between botanical drug counts and their corresponding standard deviations. For evaluating linear associations, Pearson correlation coefficients were applied to examine two key relationships: 1. Between the Dedis and DeSD indicators; 2. Between botanical drug counts and standard deviations.

## 3 Result

### 3.1 Data characteristics

The 94 filtered prescriptions from a prior study were analyzed (Supplementary Table S1). These prescriptions were derived from historical medical texts spanning the Song, Yuan, Ming, and Qing dynasties in China. After comparing outputs from non-dosage and dosage-weighted networks for each prescription, we observed that the DeSD and Dedis values - used to quantify the differences in inputs between the two types of networks - ranged from 0.12 to 34.37 and 0.35 to 99, respectively. Additionally, the DeDT and DePy indicators, which were employed to access the difference in network analysis outputs, ranged from 0.0% to 68.9% and 0.0%–74.6%, respectively, suggesting the impact of dosage across prescriptions (Supplementary Table S2).

### 3.2 Producing Dedis is key for the dosage to affect output

**FIGURE 1:**
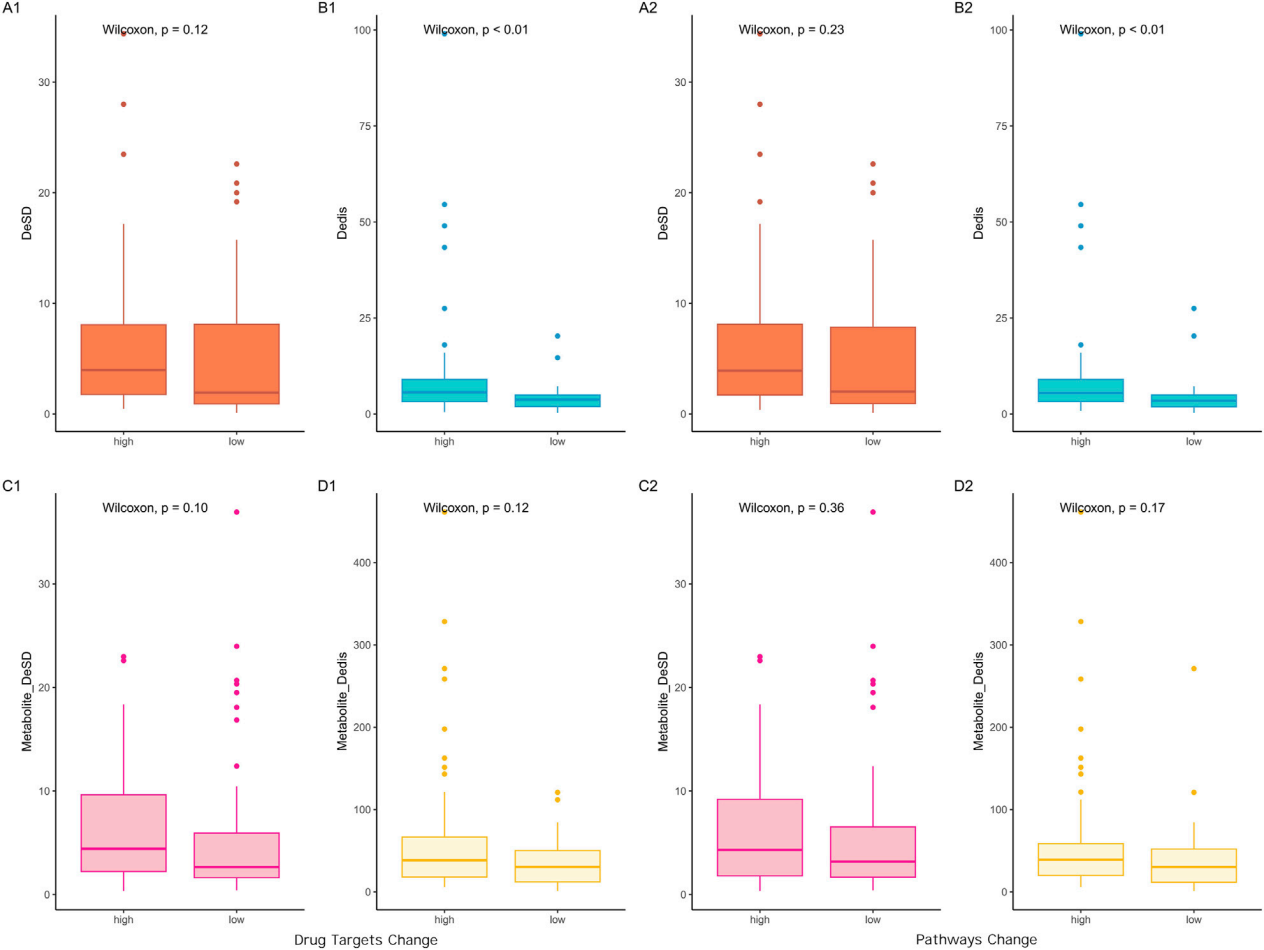
Input-output relationship analysis based on comparison between non-dosage and dosage-weighted networks. Left panels **(A1–D1)** display input indicators versus differences in predicted drug targets (DeDT); right panels **(A2–D2)** show input indicators versus pathway differences (DePy). First row: botanical drug-level analysis; second row: metabolite-level analysis. X-axis groups: Prescriptions divided by median output difference (“high” > median, “low” ≤ median; cutoff values: DeDT = 0.029, DePy = 0.068). DeSD, as one indicator of input difference, is the difference of standard deviation of the dosage of botanical drugs between non-dosage and dosage-weighted networks; Dedis, another indicator of input difference, is the Euclidean distance of the dosage of botanical drugs between two networks; The compound_DeSD and compound_Dedis are the same definition but at the metabolite level.

DeSD and Dedis were created to delineate the differences in network input attributable to dosage from two distinct perspectives. DeSD quantified the deviation of dosages from the mean value, while Dedis measured the distance between evenly distributed dosages and specific dosage values. A significant linear relationship was observed between these two indices (cor = 0.49, p = 4e-7). To evaluate the impact of incorporating dosage into the network, outcomes from networks that considered dosage were compared with those that did not, resulting in the generation of DeDT and DePy indicators. Based on the median values of DeDT and DePy, the 94 results were categorized into two groups: high output change and low output change. Upon comparing the DeSD and Dedis values between these groups, it was observed that only Dedis exhibited a statistically significant difference (Figure 1).

Botanical drugs and their metabolites serve as inputs to the network at two distinct levels. Botanical drugs typically comprise a complex mixture of metabolites, which act as the direct agents interacting with target genes, thereby determining the efficacy of the botanical drug. To further investigate the relationship between DeSD/Dedis and DeDT/DePy, a comparative analysis found that Dedis did not exhibit statistically significant difference at the metabolite level. In light of this observation, we proposed that the therapeutic effects of botanical drugs were driven by the synergistic interactions of multiple bioactive metabolites, rather than the mere summation of their individual functions. This conclusion was supported by the well-documented phenomenon that isolated so-called key metabolites from botanical drugs often failed to replicate the therapeutic effects observed in whole extracts (Gilbert and Alves, 2003; Rasoanaivo et al., 2011; Caesar and Cech, 2019).

**FIGURE 2:**
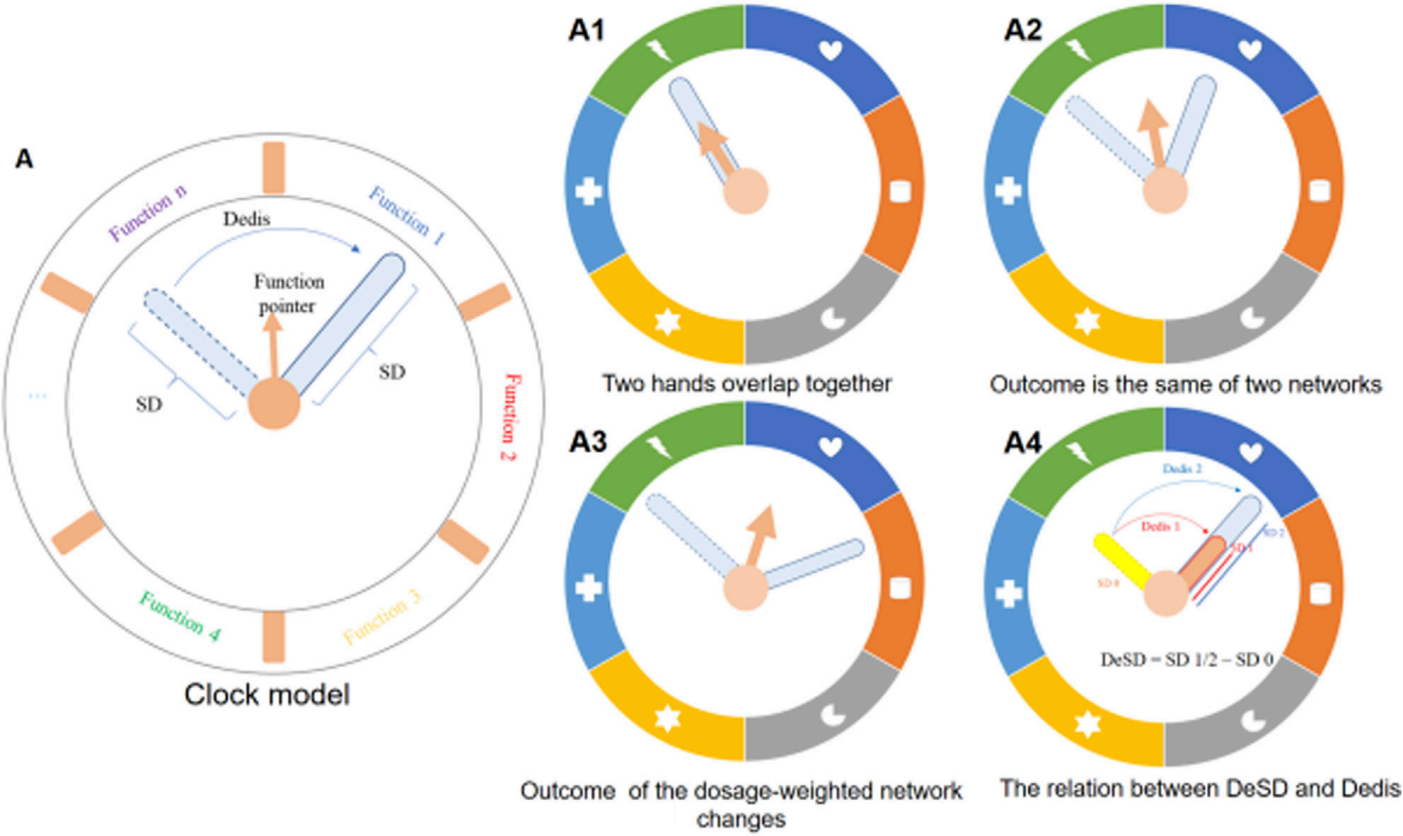
Description of the clock model. **(A)** schematic illustration of the clock model. The dosage of components was abstracted as hands on the dial, the dashed hand represented the non-dosage network and the solid outline represented the dosage-weighted network. Standard deviation (SD), an intrinsic property of the dosage vector, was abstracted as the length of the hand. The distance of the dosage of two networks (Dedis) was abstracted as an arc corresponding to the angle formed by the two hands. The arrow named function pointer denotes the output of the dosage-weighted network. **(A1–A4)** The four statuses of the clock model. **(A1)** No Dedis formed and two hands overlap. The function pointer does not change. **(A2)** The Dedis formed but it could not change the function pointer to the next function interval. **(A3)** The formed Dedis changes the function pointer to the next function interval. **(A4)** Longer solid hand produces a longer arc with the dished hand.

To better illustrate these relationships, a clock model was constructed (Figure 2A). As shown, the function pointer shifted when the two hands formed an arc, but it still pointed to the same function when the arc was not long (Figure 2A2). The arc represented the Dedis and the function pointer represented the output of the dosage-weighted network. The clock model illustrated that Dedis was a necessary and insufficient condition to determine the output change (Figure 2A1,A2,A3). In addition, the standard deviation (SD) was the intrinsic property abstracted as the length of the hands. The difference in length of the two hands cannot change the direction of the function pointer. Therefore, the DeSD should not be used as the indicator to predict whether the dosage-weighted network might yield altered output or not. When the angle between the two hands remained unchanged, the arc increased with the increased length of the hands, indicating a linear relationship between DeSD and Dedis (Figure 2A4).

From this clock model illustration, we can easily deduce that the efficacy of a drug combination will be enhanced when the dosage of each component multiplies proportionally (when the hand is extended, the original hand overlaps with the new one without changing the direction of the function pointer, meaning the function remains the same) (Figure 2A1).

### 3.3 A case study: comparison between non-dosage and dosage-weighted networks analysis of Chong He powder

To demonstrate the impact of dosage on TCM network analysis, we selected Chong He Powder from our dataset of 94 prescriptions (selection criteria: Dedis >0). This prescription, historically indicated for pox-infected patients with dryness and heat symptoms, comprises three botanical drugs with distinct dosages: realgar for 1 Liang, Bai Yao zi for 1 Qian, and Gan Cao for 1 Qian. This case study examined how dosage weight redistributed influence among the botanical drugs and refined the potential pathways, aligning with the documented clinical efficacy of the prescription.

#### 3.3.1 Dosage influence on drug target prediction of the Chong He Power

**FIGURE 3:**
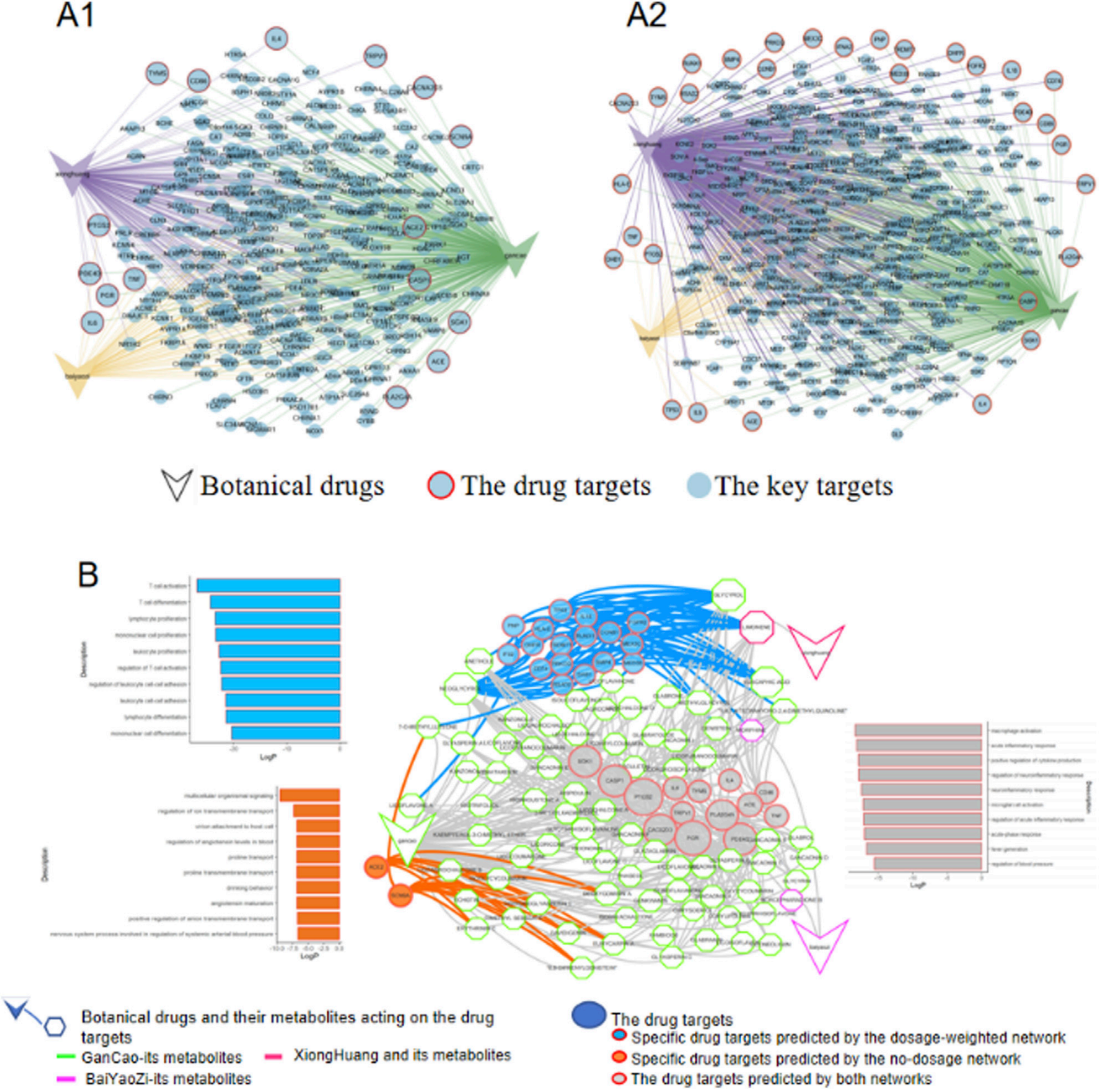
The comparison between non-dosage and dosage-weighted networks analysis of Chong He powder prescription. **(A1)** Non-dosage network predictions of key targets and drug targets; **(A2)** Dosage-weighted network predictions showing refined target selection. For clarity, bioactive metabolites bridging the botanical drugs and targets were omitted, leaving only the botanical drugs and targets shown. **(B)** Integrated target network combining predictions from both methods, with color-coded nodes indicating: (I) targets unique to the non-dosage network (orange), (ii) targets unique to the dosage-weighted network (blue), and (iii) shared targets (gray). Associated top 10 Gene Ontology pathways (ranked by significance) are displayed alongside the network.

To evaluate the impact of dosage on drug target prediction, we constructed two distinct networks for Chong He Powder: a non-dosage network and a dosage-weighted network (Figure 3A1,A2). A key finding was that the dosage-weighted network identified significantly more drug targets (represented by light-blue nodes with red outlines) compared to the non-dosage network. Notably, Realgar (xionghuang) which had a dosage ten-fold higher than the other two botanical drugs (baiyaozi and gancao), exhibited a pronounced increase in connected pox-related targets. This was evidenced by more red-outlined nodes (drug targets) linked to Realgar and increased connectivity (purple edges) in the dosage-weighted network. Since network analysis serves as a theoretical framework to elucidate the mechanistic basis of traditional prescription, a model that captures more biologically relevant targets holds greater predictive value.

#### 3.3.2 Mechanistic insights from dosage-weighted network analysis

To delineate the influence of dosage on mechanistic insights, drug targets from both non-dosage and dosage-weighted networks were integrated into a single network for better comparison (Figure 3B). The analysis revealed that most drug targets predicted by the non-dosage network were also captured by the dosage-weighted network (with gray round bubbles predominating over orange ones). These common targets were primarily related to inflammatory response and body temperature regulation, which aligns with Chong He Powder’s main efficacy of alleviating dryness and heat. However, while the non-dosage network identified pathways such as transmembrane transport and blood pressure regulation, the dosage-weighted network specifically targeted pathways focused on T cell immune regulation. Crucially, existing research strongly demonstrates that T-cell and inflammation regulation are key defense mechanisms against pox viruses (Harbour et al., 2023; Grifoni et al., 2022; Mitjà et al., 2023). In this case, the non-dosage network failed to capture this critical connection. This finding highlighted the usage of the dosage-weighted network for mechanistic elucidation in traditional medicine research.

### 3.4 Broder applications of dosage-weighted network analysis

The comparison between non-dosage and dosage-weighted network analyses in our work focused on the same prescriptions. This approach has demonstrated how integrating dosage information provides a distinct perspective for deciphering prescription mechanisms compared to the traditional non-dosage method. Importantly, the dosage-weighted network analysis can also be applied to compare different prescriptions containing identical botanical drugs but distinct dosage proportions. For example, “Gan Jie Soup” and “Ren Shen Gan Jie Soup” share the same botanical drug components but with different dosage ratios. Historical records indicated that these prescriptions from different eras were used to treat similar clinical symptoms. When applying dosage-weighted network analysis to these two formulations, identical drug targets were predicted. This finding explained how these prescriptions with varying dosages could have comparable clinical efficacy for nearly identical symptom profiles. In contrast, non-dosage network analysis would fail to reveal this dosage-efficacy relationship, as it would invariably produce identical predictions regardless of dosage variations. Therefore, dosage-weighted network pharmacology offers a valuable alternative approach to understanding combination drug principles through the lens of dosage optimization.

### 3.5 Supplemental insight: prescription dosage variability increases with fewer botanical drugs

**FIGURE 4:**
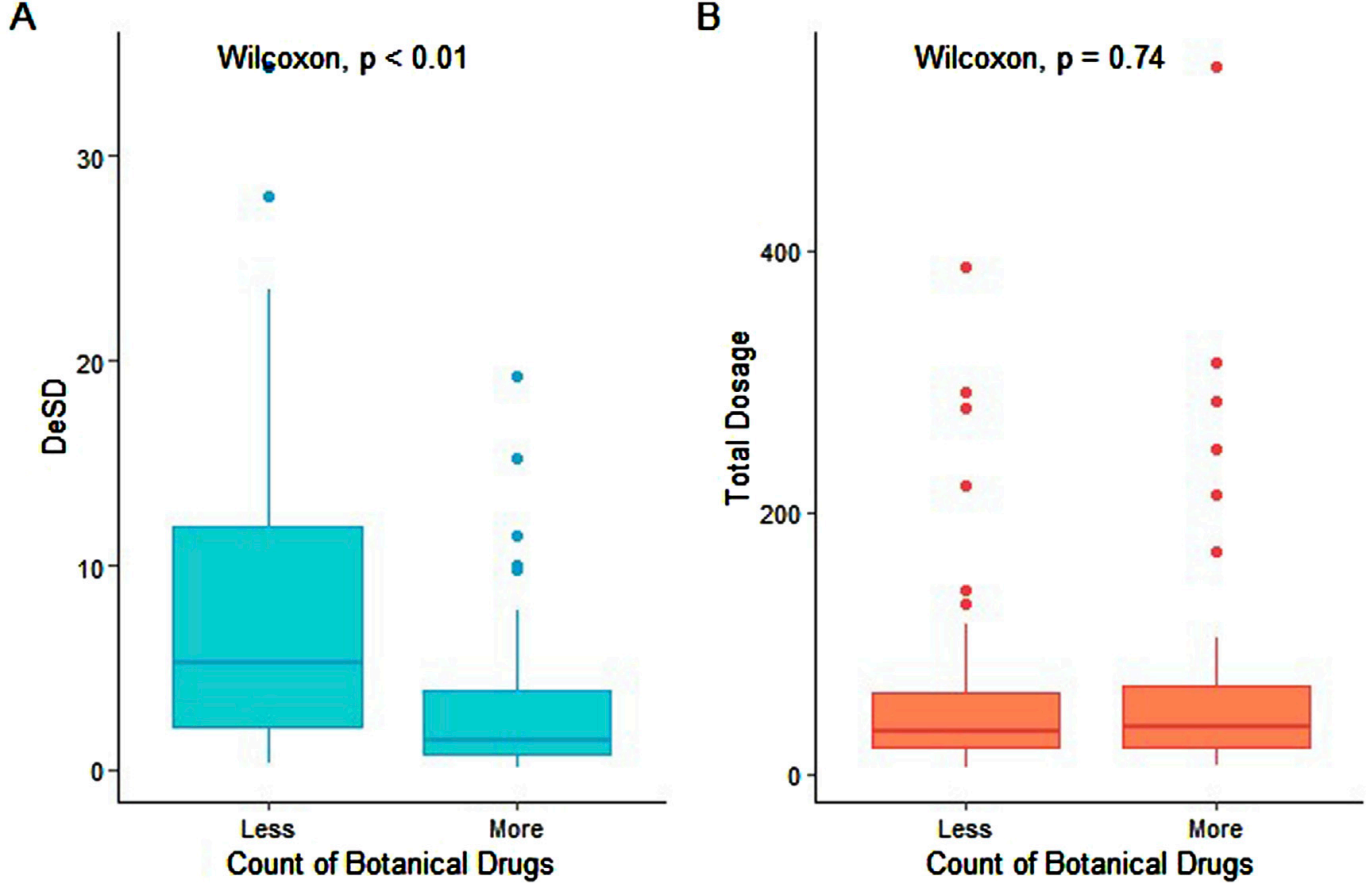
Relationship between botanical drug count and total dosage. **(A)** DeSD (difference in standard deviation) and **(B)** total dosage distributions are shown for two prescription groups: “less” (botanical drug count ≤ median 6) and “more” (count > median). Boxplot elements: center line = median; box limits = 25th–75th percentiles (IQR); whiskers = 1.5 × IQR; solid circles = outliers. Statistical comparison was performed using the Wilcoxon rank-sum test.

Secondary analysis revealed a weak inverse correlation between prescription dosage variability (standard deviation) and the number of botanical drugs (r = −0.3, p = 0.001; Figure 4A). Notably, total prescription dosage remained stable across various numbers of botanical drugs (Figure 4B). This observation may reflect established combination drug safety practices, where individual drug dosages are typically minimized to maintain overall dosage within a safe therapeutic range. Such an approach may potentially reduce the adverse effects while preserving treatment efficacy (Ali et al., 2017).

## 4 Discussion

In this study, we integrated the dosage factor into network analysis. We demonstrated that DeDis, rather than DeSD, could be used as an indicator of network prediction changes by dosage integration. Through the case study, we showed how dosage refined the impact of each botanical drug and changed the network predictions on drug targets and subsequent mechanism elucidation. Our findings suggested that dosage is a critical, yet understudied, variable in drug combination research. By integrating dosage into network analysis, a more flexible and practically accurate prediction was achieved.

Dosage is an important factor in understanding the mechanism of the drug combination and holds great potential in enhancing the usage of drug combinations. However, the cost of large-scale experiments holds back the relevant research. Some researchers used dosage data of common drug combinations to train machine-learning models for inferring appropriate dosage ranges (Zhou et al., 2021; Zhu, 2022). However, these methods often lack mechanistic explanations, producing predictions without theoretical grounding. By systematically analyzing how dosage influences drug combination efficacy, our model provided new insights into the possible mechanisms underlying dosage effect in drug combinations.

Although our analysis was conducted only on 94 TCM prescriptions, the methodology is broadly applicable to other combination drugs. A key limitation, however, is the absence of gold-standard dosage-efficacy datasets for validation. When such datasets become available, our model can be further refined to accommodate more applications.

The efficacy of complex drug combinations, such as those in TCM, depends not only on multi-component interactions but also on the dosage of individual components. Our work highlights that dosage adjustment of existing combinations is equally critical for understanding therapeutic outcomes. Moving forward, computational approaches—particularly pattern recognition and data analysis—will be essential for deciphering dosage-dependent mechanisms and advancing precision medicine in the future.

## Data Availability

The original contributions presented in the study are included in the article/Supplementary Material, further inquiries can be directed to the corresponding author.

## Abbreviations

TCM, Traditional Chinese medicine; Dedis, Difference in distance; DeSD, Difference in standard deviation; DeDT, Difference in drug target; DePy, Difference in pathways.
